# Supplementary material for: A Network Pharmacology and Multi-Omics Combination Approach to Reveal the Effect of Strontium on Ca2+ Metabolism in Bovine Rumen Epithelial Cells
Source: Int J Mol Sci. 2023 May 27;24(11):9383. doi: 10.3390/ijms24119383 (PMC10253303; doi:10.3390/ijms24119383)
Supplement: Supplementary file 1 [file ijms-24-09383-s001.zip › ijms-2348077-supplementary.pdf]

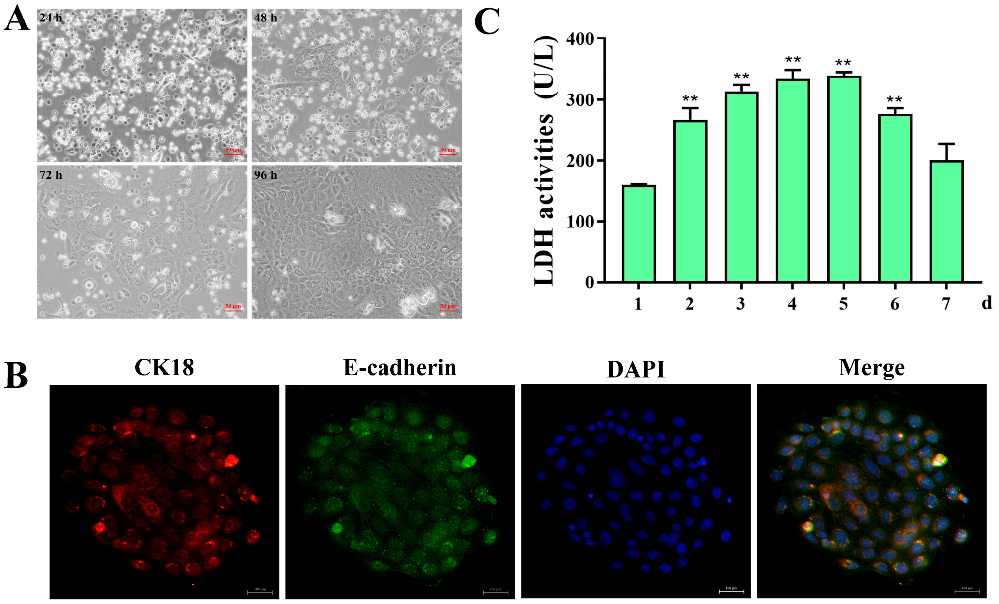

Figure S1 Culture and identification of rumen epithelial cells. (A) Cultured rumen epithelial cells. (B) Identification rumen epithelial cells by CK18 and E-cadherin positive expression. (C) LDH activity assays, the LDH activity significantly increased from day 2 to day 6.

Table S1 The results of Shapiro-Wilk test

Groups / Genes	Shapiro-Wilk test			
	0 mmol/L	1 mmol/L	10 mmol/L	20 mmol/L
CYP11A1	0.9614<br>(*P*=0.7877)	0.9525<br>(*P*=0.7316)	0.9174<br>(*P*=0.5225)	0.9412<br>(*P*=0.6618)
AHCYL2	0.8227<br>(*P*=0.1495)	0.8442<br>(*P*=0.2078)	0.9316<br>(*P*=0.6038)	0.9127<br>(*P*=0.4968)
UPK1B	0.9950<br>(*P*=0.9813)	0.9353<br>(*P*=0.6262)	0.8651<br>(*P*=0.2791)	0.8986<br>(*P*=0.4239)
WWC2	0.8753<br>(*P*=0.3189)	0.9270<br>(*P*=0.5771)	0.9864<br>(*P*=0.9384)	0.9021<br>(*P*=0.4415)
TGF-β2	0.8553<br>(*P*=0.2437)	0.8266<br>(*P*=0.1592)	0.9327<br>(*P*=0.6102)	0.8619<br>(*P*=0.2670)
SEMA3A	0.8186<br>(*P*=0.1400)	0.8613<br>(*P*=0.2647)	0.8748<br>(*P*=0.3169)	0.9290<br>(*P*=0.5887)
PTHLH	0.8756<br>(*P*=0.3202)	0.8567<br>(*P*=0.2484)	0.8685<br>(P=0.2918)	0.9038<br>(*P*=0.4502)
CA2	0.9366<br>(*P*=0.6338)	0.9199<br>(*P*=0.5364)	0.8892<br>(*P*=0.3793)	0.8638<br>(*P*=0.2740)
TGFBI	0.9004<br>(*P*=0.4330)	0.9760<br>(*P*=0.8780)	0.8054<br>(*P*=0.1123)	0.9344<br>(*P*=0.6207)
